# Analysis of Bacterial Diversity in Jersey Cow Colostrum and Mature Milk and the Study of the Probiotic Functions of *Ligilactobacillus salivarius* CR29

**DOI:** 10.1002/fsn3.70325

**Published:** 2025-05-28

**Authors:** Qibin Wang, Jianing Xu, Lichun Shang, Qingshen Sun

**Affiliations:** ^1^ Engineering Research Center of Agricultural Microbiology Technology, Ministry of Education & Heilongjiang Provincial Key Laboratory of Plant Genetic Engineering and Biological Fermentation Engineering for Cold Region & Key Laboratory of Microbiology, College of Heilongjiang Province & School of Life Sciences Heilongjiang University Harbin China; ^2^ Harbin Wonderful Jersey Raw Milk Base Co. Ltd Harbin China

**Keywords:** bacterial communities, Jersey cow, *Ligilactobacillus salivarius*, milk, probiotic properties

## Abstract

This study used 16S rDNA high‐throughput sequencing to analyze the bacterial diversity of Jersey cow colostrum and mature milk, and evaluated the probiotic properties of *Ligilactobacillus salivarius* CR29 isolated from the samples. The bacterial community structure of the Jersey cow milk samples was analyzed, and lactic acid bacteria were isolated using MRS medium. The potential probiotic strains were evaluated for growth, acid production, hemolytic activity, antibacterial activity, antibiotic sensitivity, acid and bile tolerance, hydrophobicity, autoaggregation and coaggregation abilities, as well as their survival in a simulated gastrointestinal environment. The results showed that nine lactic acid bacterial strains isolated from the milk samples exhibited probiotic potential, among which *Ligilactobacillus salivarius* CR29 showed no hemolysis and had strong antibacterial activity, significantly inhibiting several pathogens compared to other strains. CR29 was sensitive to multiple antibiotics (such as tetracycline and rifampicin), and its survival rates under 0.3% bile salt and pH 2 conditions were 56.28% and 77.59%, respectively. Its survival rate after simulated gastrointestinal passage was 47.86%. In summary, *Ligilactobacillus salivarius* CR29 demonstrated excellent probiotic potential and may be applied in functional foods and health‐related fields in the future.

## Graphical abstract

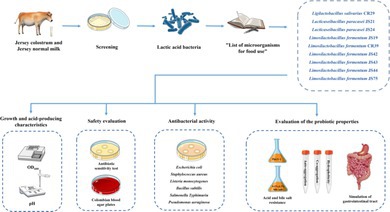

## 1 Introduction

Lactic acid bacteria (LAB) play a crucial role in the food industry and health field, and their probiotic properties are widely recognized. These microorganisms not only enhance the taste and quality of food but also positively impact human intestinal health by regulating intestinal flora, lowering cholesterol, and providing other benefits (Meng et al. 2024). They are extensively used in the fermentation industry, health foods, and microecological preparations. Consequently, the exploration of LAB and their potential applications has become a current research hotspot.

Jersey cows are renowned for their high heat tolerance (Park et al. 2021) and superior production performance. Their milk, characterized by high fat and protein content, is a valuable resource in the dairy industry (Børsting et al. 2023). Bovine colostrum, the first milk secreted after a calf's birth, is rich in high‐quality vitamins, minerals, biologically active components, and pathogen‐specific antibodies (Chae et al. 2017). It also contains numerous microorganisms, among which LAB play a critical role in maintaining intestinal microbial balance (Eshaghi et al. 2017). Upon entering the gastrointestinal tract, these microbes can adhere to intestinal epithelial cells, inhibiting pathogen attachment (Minj et al. 2020), and directly destroy pathogens through the production of lactic acid, bacteriocins, and reactive oxygen species (Rastogi and Singh 2022). Additionally, LAB can alter the intestinal microbial composition by regulating luminal pH, increasing mucus production, and secreting antimicrobial peptides, thereby enhancing barrier protection (Dempsey and Corr 2022).

For LAB to exert probiotic effects, they must withstand unfavorable conditions, including low pH and bile acids during gastrointestinal passage. They should also exhibit good adhesion capacity, autoaggregation, and surface hydrophobicity to colonize gastrointestinal epithelial cells. Furthermore, antimicrobial activities and the ability to coaggregate with pathogens are essential. Safety evaluations, such as antibiotic susceptibility testing and hemolytic activity assessment, are necessary before introducing these microorganisms into foods.

The aim of this study was to explore the bacterial diversity of Jersey colostrum and mature milk by 16S rDNA high‐throughput sequencing, and to isolate and identify LAB strains with excellent probiotic properties. We comprehensively assessed their growth characteristics, acid production capacity, bacteriostatic activity, antibiotic sensitivity, and survival in a simulated gastrointestinal environment to evaluate their potential applications for food and nutritional improvement. This work aims to provide a scientific basis and practical guidance for the development of the dairy industry and the innovation of probiotic products.

## 2 Materials and Methods

### 2.1 Reagents

de Mann Rogosa and Sharpe (MRS) broth medium (M8540, Beijing Solarbio Science & Technology Co. Ltd., Beijing, China); LB broth medium (02–136, AOBOX, Beijing, China); Columbia blood agar (Dijing Microbial Technology Co. Ltd., Guangzhou, China); amoxicillin (A6180F), tetracycline (T9020), rifampicin (R6010F), erythromycin (E6100), clindamycin (C0040F), gentamicin (G6170F), and chloramphenicol (C6050F) were purchased from Beijing Boao Tuoda Technology Co. Ltd., Beijing, China; heptadecyl salt (01–046, AOBOX, Beijing, China); ethyl acetate (A.R, Tianli Chemical Reagent Co. Ltd., Tianjin, China); artificial gastric juice and artificial intestinal juice were purchased from Shanghai Yuanye Biotechnology Co. Ltd., Shanghai, China. All the other reagents were of analytical purity.

### 2.2 Strain and Culture Conditions

The indicator bacteria used in this study were *Escherichia coli* (ATCC 25922), *Staphylococcus aureus* (ATCC 25923), *Listeria monocytogenes* (CMCC 54001), *Salmonella typhimurium* (SL.1344), *Bacillus subtilis* (ATCC 26633), and *Pseudomonas aeruginosa* (ATCC 27853), which were all preserved by the Key Laboratory of Microbiology of Heilongjiang University. The strains were grown in LB broth medium and incubated at 37°C with shaking at 120 rpm for 24 h.

### 2.3 Sample Collection and High‐Throughput Sequencing

The milk samples were provided by a farm in Harbin City, Heilongjiang Province. Jersey cows in good condition (cows with 2–5 parity) were selected in August 2023. The feed was fed with a total mixed ration, and the colostrum was collected within 2 h. Jersey colostrum labeled as JS (JS1‐JS6) and Jersey mature milk CR labeled as (CR1–CR6) were collected by the farm staff. When collecting milk, the staff first wiped the nipple with warm water to make it clean, then disinfected and wiped it with 75% alcohol, and finally, performed artificial milking. Each group of milk samples was 10 mL, placed in freezing tubes, and transported in the cold chain at low temperatures to avoid deterioration caused by high temperatures in the summer. The total genomic DNA of the milk samples was extracted, after which the purity and concentration of the DNA were detected using 1% agarose gel electrophoresis. The primers amplified the 16S V3–V4 region: 341F (5′‐CCTACGGGRSGCAGCAG‐3′) and 806R (5′‐GGACTACVVGGGTATCTAATC‐3′), and the PCR products that passed the assay were purified and the products were recovered using a DNA recovery kit for the target bands. Library construction was performed using the NEB Next➅Ultra II FS DNA PCR‐free Library Prep Kit (E7645B, New England BioLabs Inc., Ipswich, MA, USA). After the library was qualified, the PE 250 was up‐sequenced using the sequencing instruments (NovaSeq 6000; Illumina Inc., San Diego, CA, USA), and the high‐throughput sequencing process was completed by Beijing Novogene Co. Ltd.

### 2.4 Isolation and Characterization of LAB

Under sterile conditions, 1 mL of milk sample was pipetted into 9 mL of 0.85% (w/v) saline and serially diluted. A 100 μL aliquot of the appropriate dilution was spread onto MRS solid medium and incubated in a 37°C constant temperature incubator (DNP‐9082; Shanghai Jinghong Experimental Equipment Co. Ltd., Shanghai, China) for 24–48 h. The morphological characteristics of the colonies were observed. Single colonies with white or creamy smooth morphology were selected and streaked on MRS solid medium using a three‐zone line method, incubated at 37°C for 24 h, and repeated twice to obtain pure single colonies. These colonies were inoculated in MRS liquid medium and incubated overnight at 37°C with shaking at 120 rpm. The bacterial suspension was used for morphological (Gram staining) and biochemical (catalase test) analysis.

A bacterial strain testing negative for catalase and positive for Gram staining was sent to Shanghai Paiseno Biotechnology Co. Ltd. for single bacterial sequencing. Bacterial genomic DNA was extracted and subjected to PCR amplification with the following primers: 27F (5’‐AGAGTTTGATCCTGGCTCAG‐3′) and 1492R (5’‐GGTTACCTTGTTACGACTT‐3′). The PCR products from each bacterium were purified and sequenced using an ABI3730‐XL sequencing instrument (ABI3730‐XL; Applied Biosystems Inc., Carlsbad, CA, USA). The assembled sequences were compared with the 16S database for species identification.

### 2.5 Growth and Acid Production Characteristics of LAB

Strains listed in the “List of Strains Usable in Food” were analyzed for growth and acid production characteristics. They were cultivated in MRS liquid medium at 37°C for 24–48 h. The OD_600_ values and pH values were recorded every 2 h using a full‐wavelength microplate reader (SpectraMax 190; Molecular Devices LLC, Shanghai, China) and a pH meter (FE20; Mettler Toledo Instruments Co. Ltd., Shanghai, China), followed by plotting the growth and acid production curves.

### 2.6 Safety Evaluation of LAB

#### 2.6.1 Hemolysis

The hemolytic assay was performed according to the method of Kuerman et al. (2021). Strains subcultured for two generations were streaked on Columbia blood plates and incubated at 37°C for 24 h. The hemolytic activities were considered positive if a clear hemolytic zone appeared around the colonies.

#### 2.6.2 Antibiotic Sensitivity

The sensitivity of LAB to seven antibiotics (ampicillin, tetracycline, rifampicin, erythromycin, clindamycin, gentamicin, and chloramphenicol) was assessed using the agar diffusion method. The activated and passaged LAB were centrifuged at 8000 rpm for 10 min, the pellets were washed with saline twice, and adjusted to an OD_600_ of 1.0. Using the Oxford cup method, 100 μL of bacterial suspension was spread on MRS solid medium, and 200 μL of antibiotics was added to the Oxford cup wells. Plates were incubated overnight at 37°C, and results were interpreted according to the Clinical and Laboratory Standards Institute guidelines (CLSI 2023).

### 2.7 Antibacterial Activity of LAB

LAB were centrifuged at 8000 rpm for 10 min using a benchtop high‐speed centrifuge (H1850; Hunan Xiang Yi Laboratory Instrument Development Co. Ltd., Hunan, China) to collect the cell‐free supernatant, which was neutralized using 1 mol/L NaOH to eliminate acidic interference. The antibacterial activity of the LAB was determined using the agar diffusion method as described by Mulaw et al. (2019). Sterilized MRS solid medium was poured into sterile Petri dishes, and after cooling to room temperature, various indicator bacteria ( *Escherichia coli* , *Staphylococcus aureus* , *Listeria monocytogenes* , *Bacillus subtilis* , *Salmonella Typhimurium* , and *Pseudomonas aeruginosa* ) were spread on the plates. Subsequently, three sterile Oxford cups with a diameter of 8 mm were placed on the medium using sterile tweezers. After solidification, the cups were removed, and 200 μL of the neutralized cell‐free supernatant was added to each well. The plates were then incubated at 37°C for 24 h, and the diameters of the inhibition zones were measured.

### 2.8 Evaluation of the Probiotic Properties of LAB

#### 2.8.1 Acid and Bile Salt Resistance

The acid and bile salt resistance of isolated LAB was evaluated based on the method of Dbeibia et al. (2023). The isolated LAB were inoculated into MRS liquid medium with different pH values (pH 2, 3, and 4.5) and bile salt concentrations (0.1% and 0.3%), the unadjusted MRS was used as a control, and the survival rate was calculated by plate counting method after incubation at 37°C for 3 h.

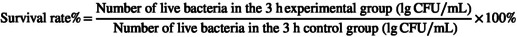




#### 2.8.2 Autoaggregation, Coaggregation, and Hydrophobicity

The autoaggregation ability of LAB was determined according to Missaoui et al. (2019). The bacterial suspensions were adjusted to *OD*_*600*_ = 1.0 (A_0_). Four milliliter of the bacterial suspension was vortexed with a vortex shaker (Shanghai Hutong Experimental Co. Ltd., Shanghai, China) for 10 s, the absorbance at 600 nm was measured after incubating at 37°C for 5 h, the *A*_*t*_ was recorded, and the autoaggregation was calculated according to the formula:

$$\text{Auto}-\text{aggregation}\%=\left(1-\frac{A_{t}}{A_{0}}\right)\times 100\%$$

The coaggregation ability of LAB with *Escherichia coli* and *Staphylococcus aureus* was determined similarly. The bacterial suspension and pathogenic bacteria were adjusted to *OD*_*600*_ = 1.0, respectively, which were recorded as *A*_*0*_. 2 mL of bacterial suspension was mixed with 2 mL of each pathogenic bacterial suspension, vortexed for 10 s, incubated at 37°C for 5 h, and then the absorbance at 600 nm (A_x_) was measured and the coaggregation was calculated according to the formula:

$$\mathrm{Co}-\text{aggregation}\%=\left[\frac{\left(A_{0}+A_{t}\right)-2\times A_{x}}{A_{0}+A_{t}}\right]\times 100\%$$

The hydrophobicity of LAB was determined using ethyl acetate according to Dbeibia et al. (2023) with slight modifications. The bacterial suspension was adjusted to *OD*_*600*_ = 1.0 (recorded as *A*_*0*_); 1 mL of organic solvent was mixed with 3 mL of bacterial suspension, vortexed for 2 min, and then stood for 30 min to measure the absorbance *A*_*x*_ at 600 nm by absorbing the aqueous phase and the hydrophobicity was calculated according to the formula:

$$\;\text{Hydrophobicity}\%=\left(1-\frac{A_{x}}{A_{0}}\right)\times 100\%$$

#### 2.8.3 Gastrointestinal Stability Analysis

The LAB were subjected to a simulated gastrointestinal test according to Qi et al. (2024) After the LAB were activated and passaged twice, 1 mL of bacterial suspension (*OD*_*600*_ = 1.0) and 5 mL of artificial gastric juice were mixed and incubated at 120 rpm in a 37°C shaker for 2 h. Subsequently, the bacterial suspension was centrifuged at 8000 rpm for 15 min, followed by washing with saline. The LAB treated with artificial gastric juice were cultured on MRS solid medium, and the number of viable bacteria was counted. Then, the treated bacterial sludge was placed in 5 mL of artificial bile and incubated at 120 rpm in a shaker at 37°C for 20 min, and the number of viable bacteria was counted as described above. Finally, the bile‐treated bacterial sludge was added to 5 mL of artificial intestinal fluid (medium), incubated at 120 rpm in a shaker at 37°C for 2 h, and the number of viable bacteria was counted as described above.

### 2.9 Data Analysis

All experiments were conducted in triplicate, the data were statistically analyzed and graphed using Origin 9.0 software (Origin Lab Corporation, Northampton, MS, USA), the numerical results were expressed as $\bar{X}$ ± SD standard deviation, and the significance level was *p* < 0.05.

## 3 Results and Discussion

### 3.1 16S rDNA High‐Throughput Sequencing Results

**FIGURE 1:**
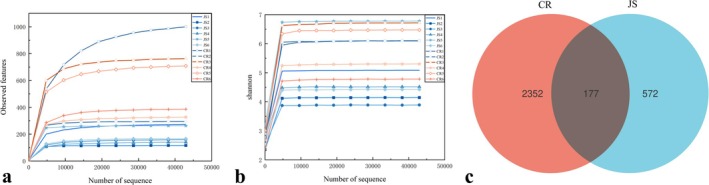
Rarefaction curves for observed features (a) and Shannon index (b), and a Venn diagram of sample OTUs distribution (c). Abbreviations: CR, Jersey mature milk group; JS, Jersey colostrum group.

In this study, 16S rDNA high‐throughput sequencing technology was used to compare and analyze the composition and diversity of microorganisms in colostrum and mature milk of Jersey cows. It can be seen from Figure 1a,b that the dilution curves of all milk samples reached the platform period, indicating that the sequencing depth met the requirements of subsequent analysis. A total of 2529 OTUs were produced in the CR group, while 749 OTUs were produced in the JS group, of which 177 OTUs were produced in both groups (Figure 1c). It can be seen that the number of OTUs in the mature milk group was much higher than that in the colostrum group, indicating that the bacterial diversity in the mature milk group was higher than that in the colostrum group.

**FIGURE 2:**
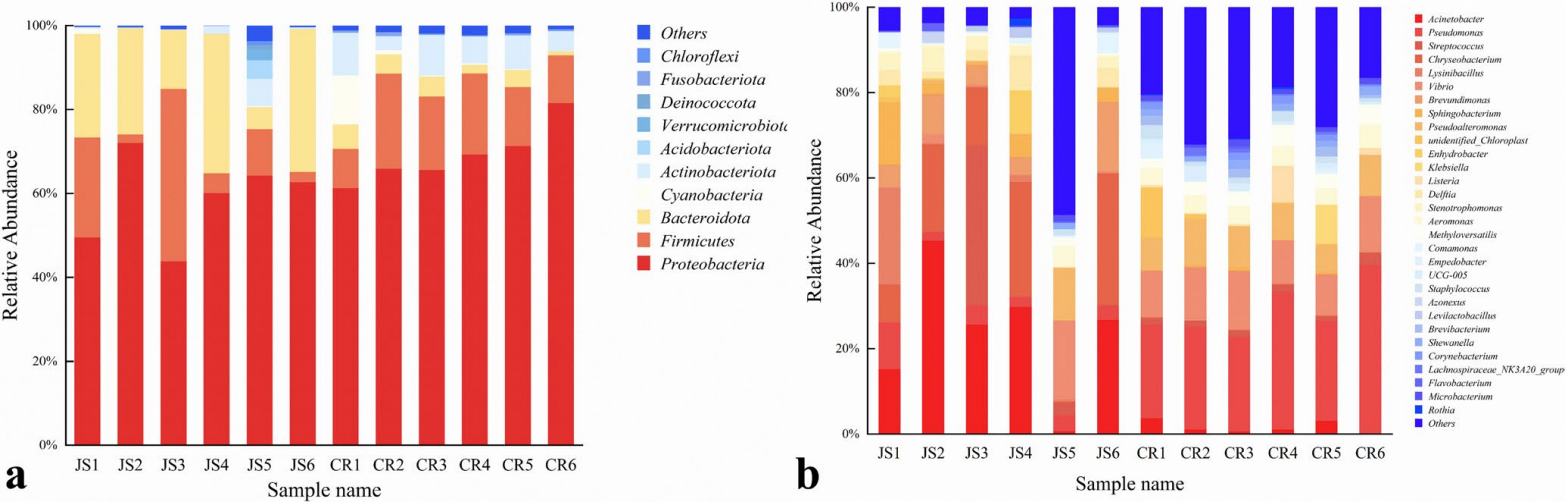
Relative abundances of the microbiota of Jersey milk at the phylum (a) and genus (b) level.

At the phylum level, the core flora in colostrum and mature milk consisted of *Proteobacteria*, *Firmicutes*, *Bacteroidetes*, and *Actinobacteriota* (Figure 2a), consistent with the previous findings (Breitenwieser et al. 2020). *Bacteroidetes* was substantially more abundant in JS milk (22.7%) than in CR milk (3.7%). Conversely, Actinobacteriota showed higher proportions in CR milk (7.1%) compared to JS milk (1.5%) (Figure 2a).

**FIGURE 3:**
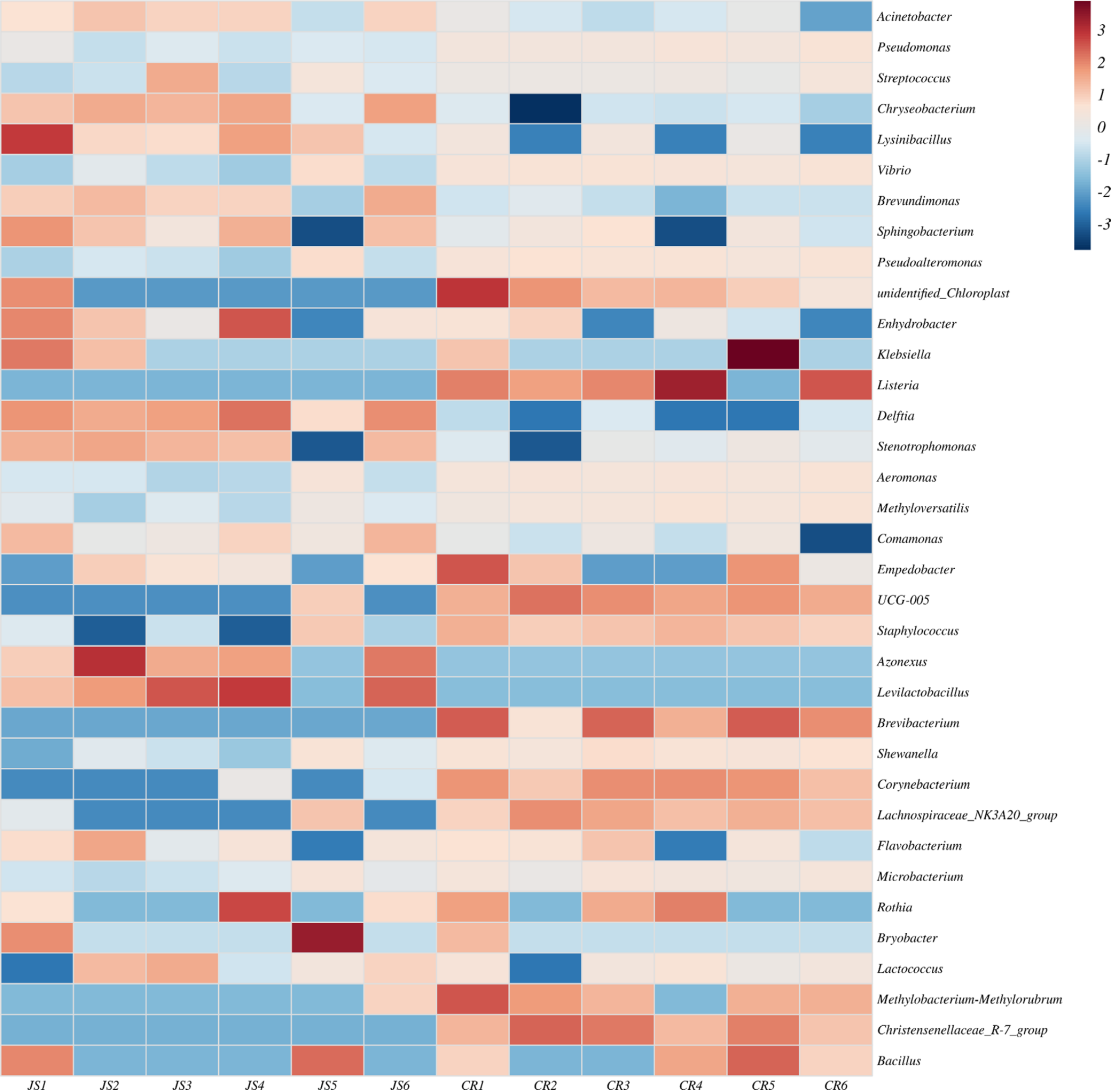
Community clustering heatmaps of samples based on genus level. The upper right legend in the figure is the value represented by the color gradient. The redder the color in the figure indicates the higher the abundance of the colony, and the bluer the color indicates the lower the abundance of the colony in the sample.

At the genus level, significant compositional differences were observed between colostrum and mature milk (Figure 2b). The colostrum microbiota was dominated by *Acinetobacter* and *Chryseobacterium*, which could also be observed from Heatmap analysis (Figure 3), both of which revealed anti‐infection capabilities (Xie et al. 2021a); while the dominant flora in the mature milk group was *Pseudomonas*, *Pseudoalteromonas*, and *Vibrio*. The proportion of *Acinetobacter* in the colostrum group was much higher than that in the mature milk group, and the proportion of *Vibrio* in each mature milk sample was higher. It is worth noting that the proportion of *Pseudomonas* in each sample of the mature milk group is more than 20% (Figure 2b). As a lactose‐utilizing genus, *Acinetobacter* was found in much higher proportions in colostrum compared to mature milk, and its prevalence decreases as the lactose content in mature milk samples decreases (Chen et al. 2018). Acinetobacter was sensitive to various antibiotics, which also helped in maintaining the health of dairy cows (Zhu et al. 2023). The abundance of microbial communities in cow's milk is also influenced by seasonal and environmental factors (Oikonomou et al. 2020). The samples for this study were collected during the summer, which might cause significantly higher abundance of *Acinetobacter* and *Chryseobacterium* in cow's milk, as higher temperatures in summer will also increase the risk of bacterial contamination during milk collection (Xie et al. 2021b). These results were also in agreement with another study (Gathinji et al. 2023). These findings offer new insights into the hygiene measures for managing Jersey cow farming. The *Pseudoalteromonas* abundance in the mature milk group was significantly increased as compared with that of the colostrum group (Figure 2b), which was in agreement with a previous study (Quigley et al. 2013) and Li et al. (2018). The high‐fat and high‐protein characteristics of Jersey milk led to a relatively higher abundance of *Pseudomonas*, which was consistent with the results of another study (Nguyen et al. 2020).

**FIGURE 4:**
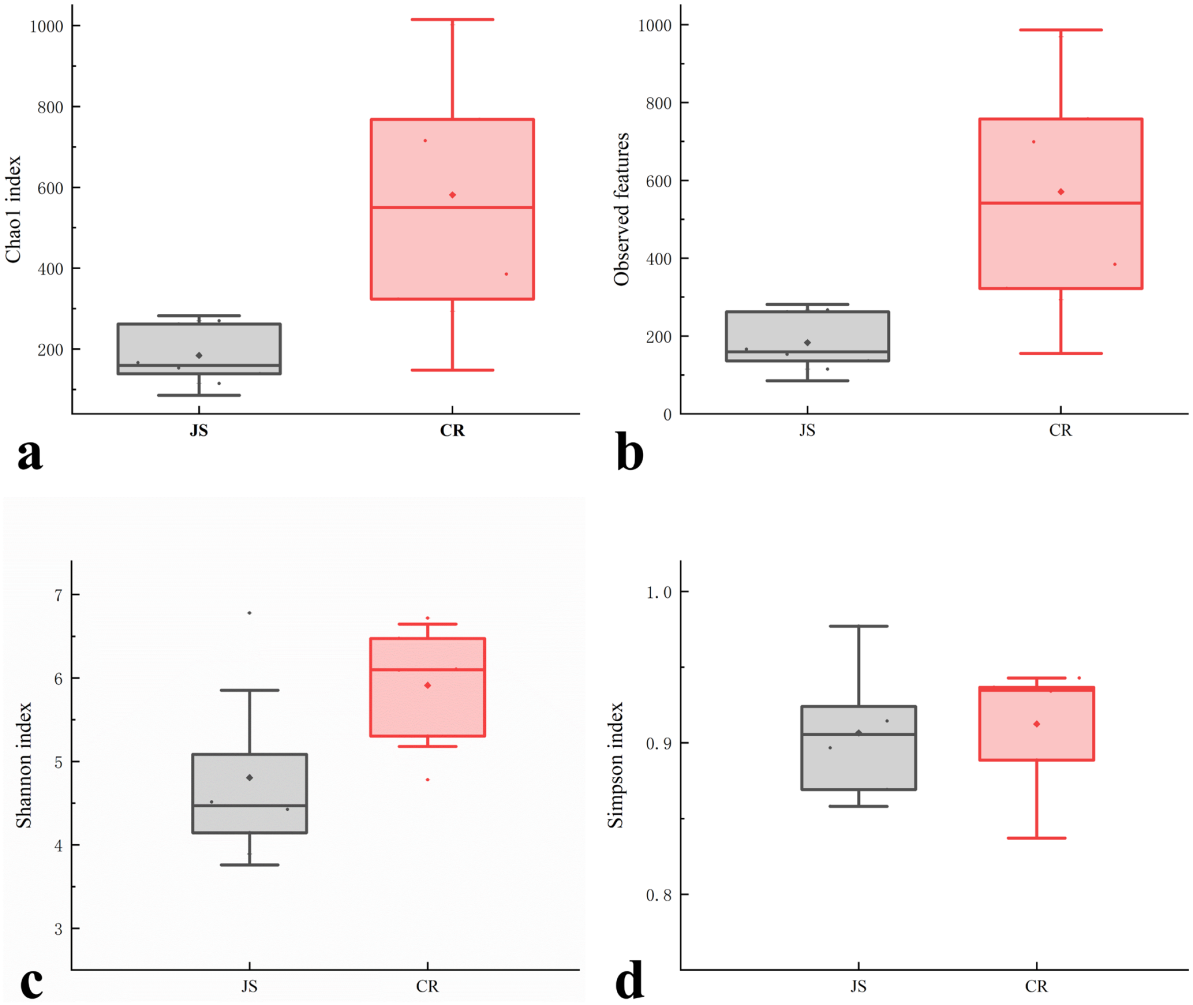
Boxplots of *α*‐diversity indexes of Jersey milk samples.

In order to reflect the diversity and evenness of species distribution, several different α‐diversity indices were used (Figure 4), and there were significant differences in observed features (Figure 4a) and Chao1 index (Figure 4b) between colostrum and mature milk samples (*p* < 0.05), indicating that the microbial richness of mature milk samples was much higher than that of colostrum samples. However, no significant difference was found in Shannon and Simpson indices between JS and CR groups (Figure 4c,d). The Shannon index (reflecting both species' richness and evenness) and Simpson index (emphasizing dominance of abundant species) consistently demonstrated that the JS and CR groups exhibited similar overall microbial diversity levels. Despite abundance variations in specific genera (e.g., *Acinetobacter* or *Pseudomonas*, as seen in Figure 2a), the overall diversity framework of the communities likely remained dynamically balanced due to combined regulation by host physiology (e.g., cattle breed and lactation stage) and environmental factors.

**FIGURE 5:**
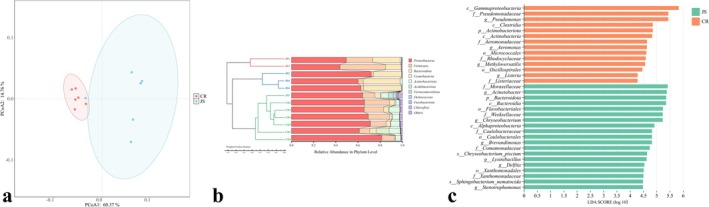
Weighted UniFrac principal coordinates analysis (PCoA) (a), UPGMA dendrogram based on Weighted Unifrac distance (b), and LDA score distribution histogram (c).

PCoA revealed distinct clustering patterns between colostrum and mature milk microbiota (Figure 5a). The first two principal coordinates accounted for 60.37% (PCoA1) and 14.76% (PCoA2) of the total variation, respectively. While a minor overlap between groups suggested partial structural similarity, mature milk samples exhibited high compositional homogeneity, whereas colostrum samples showed greater variability. UPGMA clustering further supported these trends (Figure 5b). Samples (e.g., CR3 and CR4) clustered tightly, indicating high similarity. Greater heterogeneity was observed, with JS4, JS6, and JS2 forming one subcluster, while JS1 and JS3 grouped separately. These findings are consistent with Lima et al. (2017), highlighting stage‐dependent microbial dynamics. LEfSe analysis identified 19 differentially abundant taxa in colostrum and 14 in mature milk (Figure 5c). Notably, *Moraxellaceae* significantly influenced colostrum microbiota structure, aligning with Yasir et al. (2024).

**FIGURE 6:**
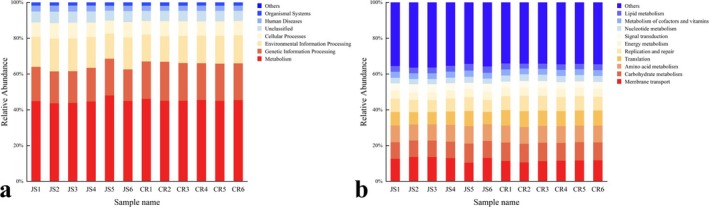
KEGG Level 1 (a) and Level 2 (b) function prediction.

In addition, Jersey milk exhibited higher relative abundance in three key functional categories: metabolism, genetic information processing, and environmental information processing (Figure 6a). KEGG functional prediction further revealed the top 10 metabolic‐related functional pathways with the highest abundance (Figure 6b), which were ranked by abundance: membrane transport, carbohydrate metabolism, amino acid metabolism, translation, replication and repair, energy metabolism, signal transduction, nucleotide metabolism, metabolism of cofactors and vitamins, and lipid metabolism. These findings align with previous research by Liu et al. (2021).

### 3.2 Isolation and Identification of LAB

#### 3.2.1 Strain Isolation Results

**FIGURE 7:**
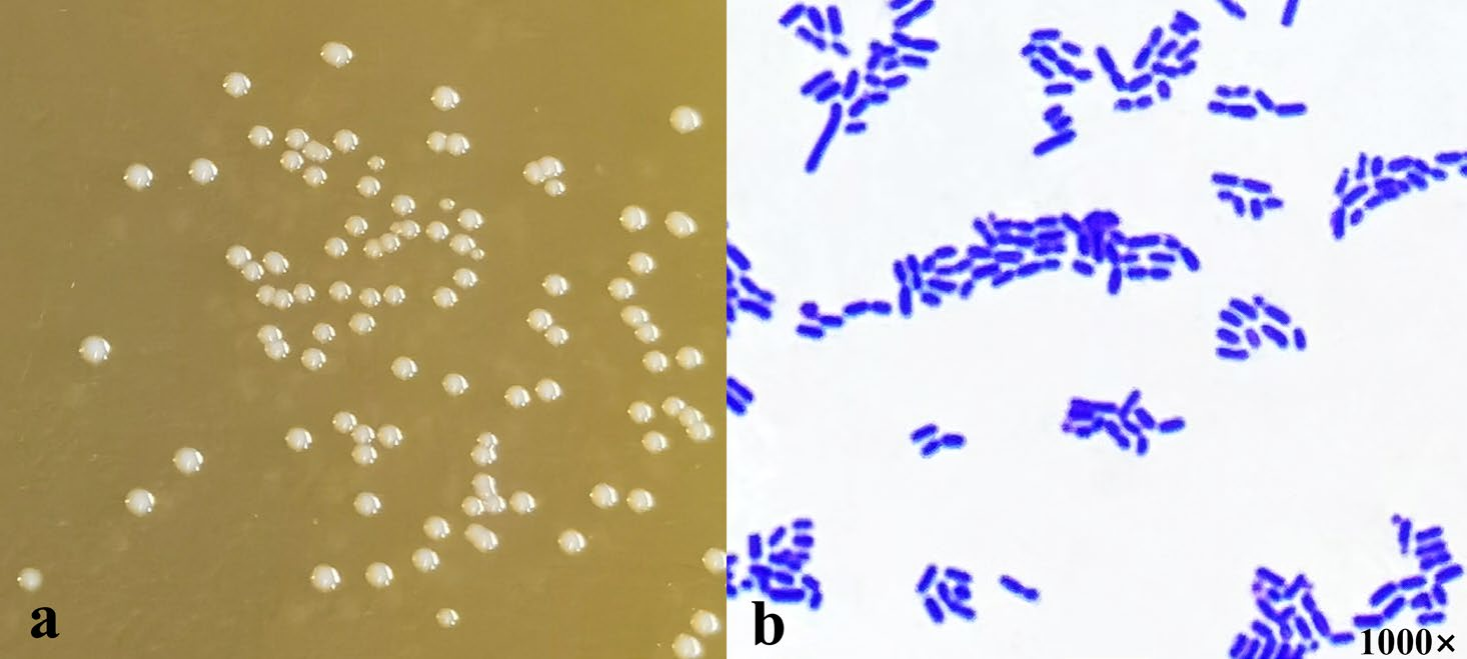
Morphological characteristics (a) and Gram staining (b) results of strain CR29 (1000×).

Preliminary screening identified 62 strains that were catalase‐negative and Gram‐positive (data not shown). Using *Ligilactobacillus salivarius* CR29 (PQ285082.1) as an example (referred to as CR29 hereinafter), the morphological characteristics of the single colonies screened on MRS solid medium (Figure 7a) and the results of the Gram staining (Figure 7b) showed that the bacterial cells had a smooth, round surface and were white or creamy.

#### 3.2.2 Strain Identification Results

The isolated strains were subjected to 16S rDNA sequencing, and the identification results were obtained through NCBI BLAST sequence alignment (Table 1). *Lactococcus garvieae* was isolated from both Jersey colostrum and Jersey mature milk, *Lacticaseibacillus paracasei* was mainly isolated from Jersey colostrum samples, and *Ligilactobacillus salivarius* was mainly isolated from Jersey mature milk. Other isolated strains were found across various samples. Based on the “List of Strains Usable in Food,” a total of nine LAB were selected for further study of their probiotic properties: six strains of *Limosilactobacillus fermentum*, two strains of *Lacticaseibacillus paracasei*, and one strain of *Ligilactobacillus salivarius*.

**TABLE 1:** Species identification results.

Number	Identification result	Number	Identification result
JS02	*Enterococcus faecalis*	JS19	*Limosilactobacillus fermentum*
JS03	*Enterococcus faecalis*	JS42	*Limosilactobacillus fermentum*
JS04	*Enterococcus faecalis*	JS43	*Limosilactobacillus fermentum*
JS2	*Enterococcus faecalis*	JS44	*Limosilactobacillus fermentum*
JS17	*Enterococcus faecalis*	JS75	*Limosilactobacillus fermentum*
JS25	*Enterococcus faecalis*	JS58	*Weissella cibaria*
JS59	*Enterococcus faecalis*	JS68	*Weissella cibaria*
JS60	*Enterococcus faecalis*	JS64	*Kurthia gibsonii*
JS61	*Enterococcus faecalis*	JS67	*Enterococcus faecium*
JS63	*Enterococcus faecalis*	JS70	*Enterococcus faecium*
JS65	*Enterococcus faecalis*	CR33	*Bacillus tropicus*
JS66	*Enterococcus faecalis*	CR35	*Enterococcus faecalis*
JS69	*Enterococcus faecalis*	CR28	*Lactococcus garvieae*
JS71	*Enterococcus faecalis*	CR30	*Lactococcus garvieae*
JS72	*Enterococcus faecalis*	CR32	*Lactococcus garvieae*
JS73	*Enterococcus faecalis*	CR39	*Limosilactobacillus fermentum*
JS05	*Lactococcus garvieae*	CR26	*Staphylococcus simulans*
JS1	*Lactococcus garvieae*	CR27	*Staphylococcus simulans*
JS21	*Lacticaseibacillus paracasei*	CR38	*Staphylococcus simulans*
JS24	*Lacticaseibacillus paracasei*	CR29	*Ligilactobacillus salivarius*

### 3.3 Growth and Acid‐Producing Characteristics

**FIGURE 8:**
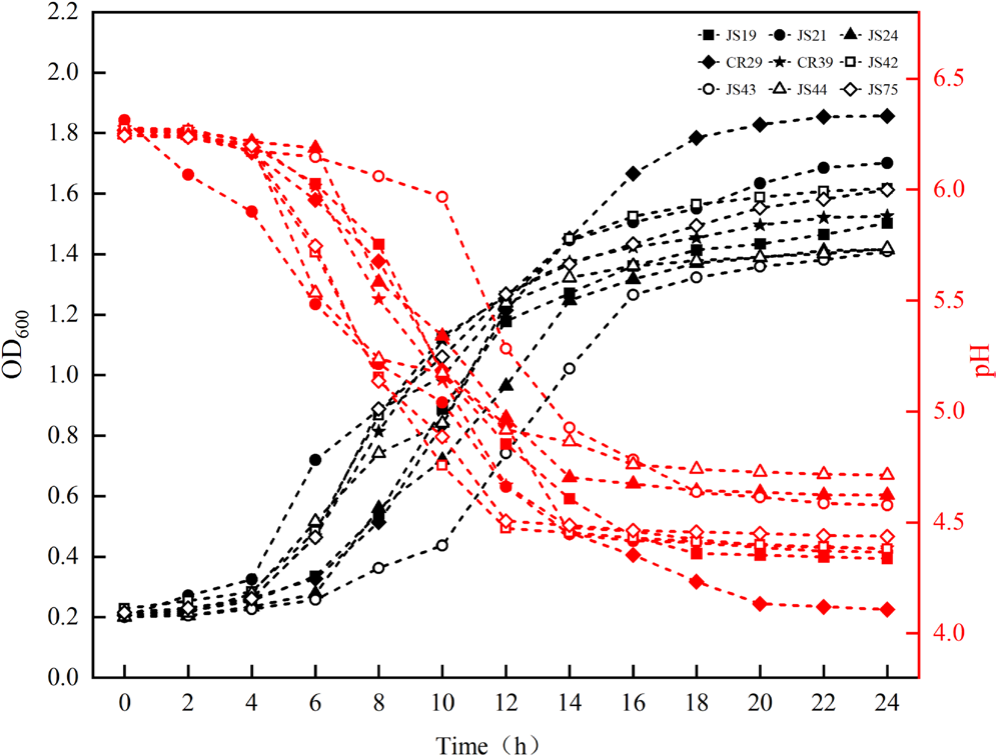
The growth and acid production curve of nine strains of lactic acid bacteria.

The growth and pH curves of the nine LAB were shown in Figure 8. Most of the strains grew rapidly within 6–16 h, which was designated as their logarithmic growth period, and then entered the stationary phase. The pH of the MRS broth decreased rapidly between 8 and 16 h and stabilized around 4.38 after the logarithmic growth period. The OD_600_ and pH of CR29 reached 1.86 ± 0.03 and 4.11 ± 0.012 at 24 h, respectively, showing good growth and acid production performance, which was the same as that of Piwat et al. (2011). *Ligilactobacillus salivarius* is a well‐characterized lactic acid bacterium, often isolated from the gastrointestinal tract of humans, porcines, and poultry, human milk, and other sources. This strain can modulate the gut microbiota, stimulate protective immune responses, and inhibit fecal enzyme activity, thereby allowing proper acidification of the gut (Messaoudi et al. 2013).

### 3.4 Safety Evaluation of LAB

#### 3.4.1 Hemolysis

**FIGURE 9:**
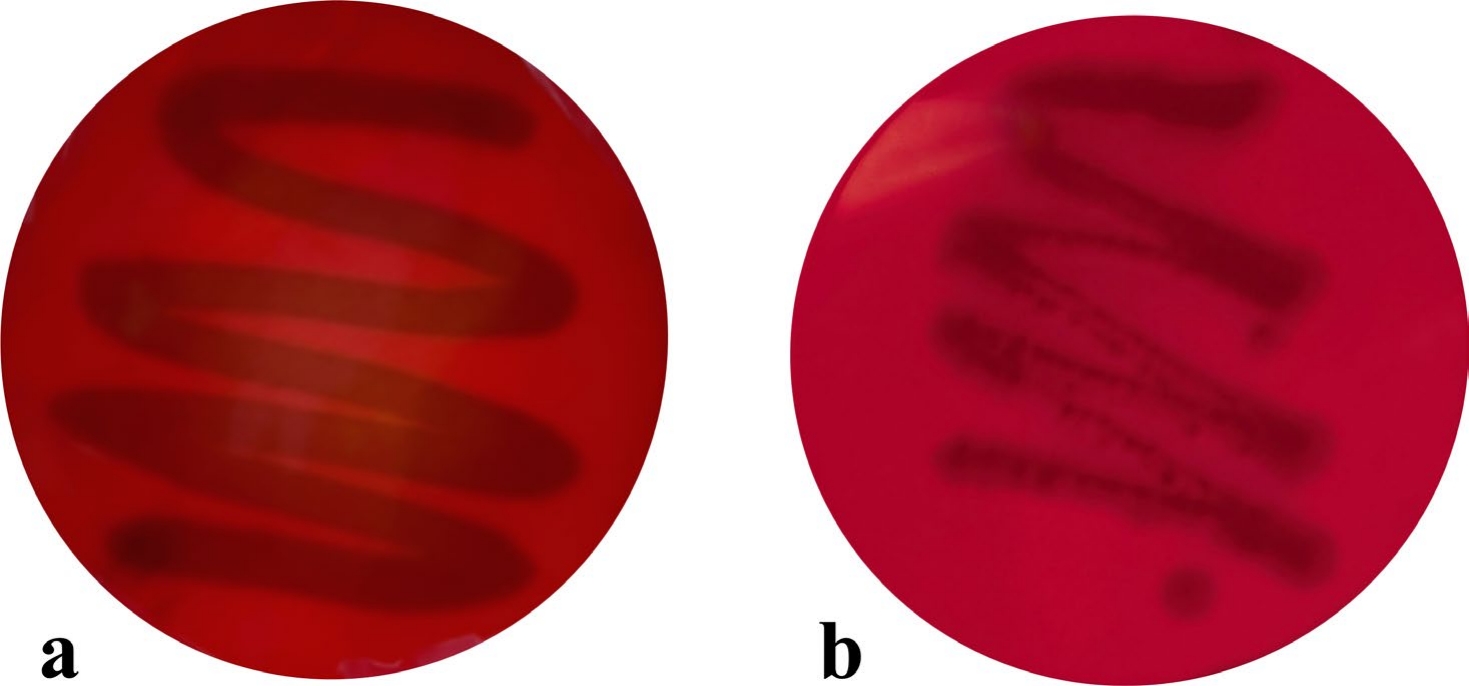
Hemolysis results of strain CR29 (a) and *Staphylococcus aureus* (b).

Safety is an important prerequisite for the use of probiotics in the food industry. Hemolytic activity was used to evaluate the safety of probiotics (Jose et al. 2015). Figure 9 displays representative images of CR29 (a) and *Staphylococcus aureus* (b), with the latter exhibiting β‐hemolysis as a positive control. None of the nine LAB strains, including CR29, showed hemolysis, indicating they were safe strains.

#### 3.4.2 Antibiotic Sensitivity

If a strain carries transferable resistance genes, it can pose a risk to the host's health. Therefore, assessing the antibiotic resistance of probiotic strains is a crucial step in the selection of potential probiotics (Abdou and Awad 2024). Table 2 shows the results of the agar diffusion method used to determine the sensitivity of nine LAB strains to seven different antibiotics. The antibiotic resistance of these nine strains exhibited interspecies similarities, with all strains showing resistance to aminoglycoside antibiotics. However, their sensitivity to other classes of antibiotics varied. CR29 was sensitive to tetracycline, ampicillin, chloramphenicol, rifampin, and clindamycin, which is similar to the findings of Langa et al. (2012), where *Lactobacillus salivarius* CECT5713, isolated from breast milk, also showed sensitivity to most of the tested antibiotics. There were also intraspecies similarities in antibiotic sensitivity, as *Lacticaseibacillus paracasei* JS21 (PQ285080.1) and JS24 (PQ285081.1) (hereinafter referred to as JS21 and JS24) were both sensitive to rifampin and exhibited moderate resistance to tetracycline. However, intraspecies differences were also observed: *Limosilactobacillus fermentum* CR39 (PQ285083.1), JS42 (PQ285084.1), and JS75 (PQ285087.1) (hereinafter referred to as CR39, JS42, and JS75) were sensitive to clindamycin, while *Limosilactobacillus fermentum* JS19 (PQ285079.1), JS43 (PQ285085.1), and JS44 (PQ285086.1) (hereinafter referred to as JS19, JS43, and JS44) exhibited moderate resistance.

**TABLE 2:** The results of antibiotic resistance of nine strains of lactic acid bacteria.

Antibiotic category	Antibiotic name	JS19	JS21	JS24	CR29	CR39	JS42	JS43	JS44	JS75
β‐Lactams	Ampicillin	S	S	S	S	S	S	S	S	S
Tetracycline class	Tetracycline	S	I	I	S	S	S	I	S	S
Macrolides	Erythromycin	I	I	I	I	I	I	I	I	I
Quinolones	Rifampicin	I	S	S	S	I	I	I	I	I
Aminoglycosides	Gentamicin	R	R	R	R	R	R	R	R	R
Other	Chloramphenicol	S	S	S	S	S	S	S	S	S
	Clindamycin	I	S	S	S	S	S	I	I	S

Abbreviations: I: intermediate; R: resistant; S: sensitive.

### 3.5 Antibacterial Activity

LAB can exert antagonistic effects by competing with pathogens for growth space, thereby inhibiting their growth (Sirikhwan et al. 2011). In this study, cell‐free supernatants (CFS) obtained after acid discharge were used to assess the antibacterial activity of the strains. The diameters of the inhibition zones are shown in Table 3. CR29 demonstrated significantly higher inhibitory activity against *Escherichia coli* , *Staphylococcus aureus* , *Listeria monocytogenes* , *Pseudomonas aeruginosa* , and *Salmonella typhimurium* compared to other strains (*p* < 0.05). Similar results were observed for *Ligilactobacillus salivarius* XP132 and *Ligilactobacillus salivarius* CNU1334 (He et al. 2024; Kang et al. 2017). Additionally, in this study, *Limosilactobacillus fermentum* CR39 exhibited significant inhibitory effects against five pathogenic strains. According to previous researchers, LAB can achieve antibacterial effects by producing organic acids, antimicrobial peptides, or bacteriocins (Rodríguez‐Sojo et al. 2021). In the current study, we have not yet determined which product affects the antibacterial ability of the experimental strain, which needs further experiments to prove.

**TABLE 3:** Antimicrobial zone diameter of tested strains against pathogens.

Strains	*Escherichia coli*	*Staphylococcus aureus*	*Listeria monocytogenes*	*Pseudomonas aeruginosa*	*Salmonella typhimurium*	*Bacillus subtilis*
JS19	13.8 ± 0.340^b^	21.0 ± 0.852^a^	20.2 ± 0.287^b^	12.2 ± 0.377^d^	14.9 ± 0.170^b^	16.2 ± 0.216^a^
JS21	12.4 ± 0.125^c^	12.8 ± 0.141^b^	13.4 ± 0.125^d^	13.3 ± 0.163^c^	10.7 ± 0.125^d^	11.3 ± 0.125^d^
JS24	13.2 ± 0.205^bc^	13.7 ± 0.245^b^	14.7 ± 0.330c	14.9 ± 0.205^b^	12.6 ± 0.094^c^	10.9 ± 0.216^d^
CR29	15.5 ± 0.205^a^	22.0 ± 0.850^a^	21.5 ± 0.589^a^	19.8 ± 0.249^a^	17.4 ± 0.216^a^	13.1 ± 0.094^b^
CR39	15.8 ± 0.189^a^	21.7 ± 0.368^a^	19.5 ± 0.205^b^	19.3 ± 0.492^a^	17.6 ± 0.236^a^	15.3 ± 0.125^a^
JS42	12.7 ± 0.356^c^	13.9 ± 0.287^b^	14.4 ± 0.294^cd^	14.5 ± 0.125^b^	10.8 ± 0.287^d^	11.9 ± 0.170^cd^
JS43	12.6 ± 0.141^c^	13.4 ± 0.236^b^	15.0 ± 0.216^c^	11.7 ± 0.386^de^	9.6 ± 0.170^e^	9.5 ± 0.309^e^
JS44	12.8 ± 0.294^bc^	14.1 ± 0.535^b^	15.6 ± 0.094^c^	10.9 ± 0.216^e^	12.9 ± 0.170^c^	12.8 ± 0.377^bc^
JS75	12.4 ± 0.094^c^	13.9 ± 0.262^b^	11.2 ± 0.082^e^	11.9 ± 0.170^d^	8.8 ± 0.047^e^	11.2 ± 0.245^d^

*Note:* Different letters of the same column data indicated significant difference (*p* < 0.05), and the same letter label indicated that the difference was not significant (*p* > 0.05).

### 3.6 Evaluation of the Probiotic Properties of LAB

#### 3.6.1 Acid and Bile Salt Resistance

**FIGURE 10:**
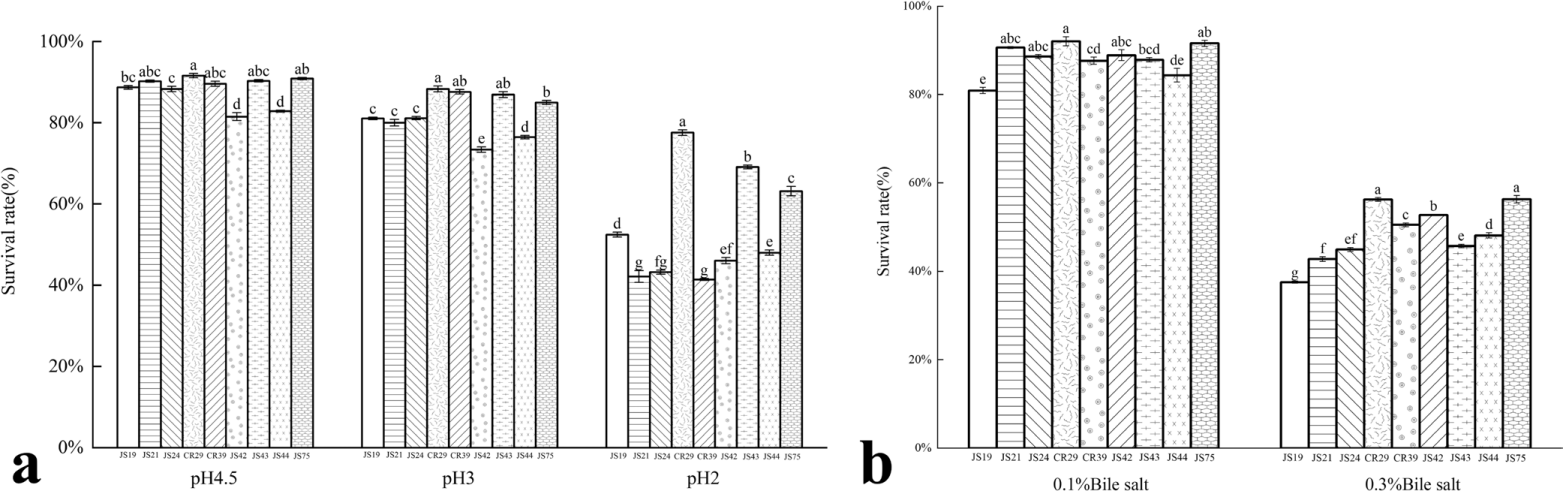
The tolerance of nine strains of lactic acid bacteria to acid (a) and bile salts (b). Different lowercase letters indicated significant differences in tolerance under the same concentration of acid and bile salt environment (*p* < 0.05) (*n* = 3).

As beneficial microorganisms in the gut, probiotics can exert a probiotic effect after colonization (Ma et al. 2023), but various environments in the gastrointestinal tract, such as strong acidic gastric juice, bile salts, and various digestive enzymes, can be lethal to them (Zhou et al. 2024), so probiotics must have the ability to resist low pH and high bile salt concentrations. The acid tolerance of the nine LAB strains was analyzed by measuring their survival rates after 3 h of growth in a low pH environment (Figure 10a). All strains exhibited relatively high survival rates at pH 4.5 and 3, with CR29 showing survival rates of 91.61% ± 0.50% and 88.33% ± 0.70%, respectively, significantly higher than those of the other strains (*p* < 0.05). At pH 2, survival rates varied among the strains, ranging from 41.40% ± 0.20% to 77.59% ± 0.70%, with CR29 demonstrating the strongest acid tolerance (*p* < 0.05).

The bile salt tolerance of the nine LAB strains was shown in Figure 10b. After 3 h of cultivation in an environment with 0.1% bile salts, all strains exhibited relatively high survival rates. However, in a 0.3% bile salt environment, their growth was inhibited, with survival rates below 60%. The highest survival rates were observed in JS75 and CR29, with 56.31% ± 0.80% and 56.28% ± 0.40%, respectively. In this experiment, CR29 had a high survival rate after being exposed to pH 2 and 0.3% bile salt concentrations, which was the same as the results of Messaoudi et al. (2012), who found that *Ligilactobacillus salivarius* SMXD51 had good acid and bile salt tolerance. Pinchao et al. (2024) isolated multiple strains of LAB from the guinea pig intestine and found that *Ligilactobacillus salivarius* had good tolerance.

#### 3.6.2 Autoaggregation, Coaggregation, and Hydrophobicity

**FIGURE 11:**
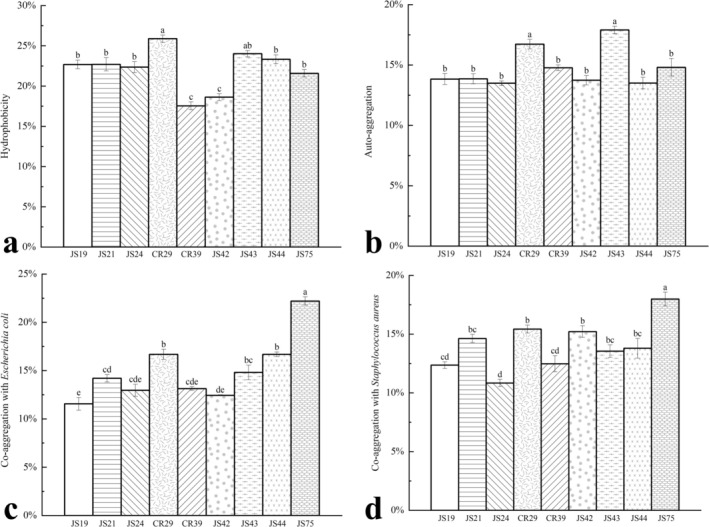
Hydrophobicity (a), autoaggregation (b), and coaggregation with *Escherichia coli* (c) and *Staphylococcus aureus* (d) of nine strains of lactic acid bacteria. Different letters of the same column data indicate significant difference (*p* < 0.05) (*n* = 3).

Strains with high hydrophobicity typically exhibit strong adhesion properties, which also determine the ability of probiotics to bind to receptors on the intestinal mucosal surface (Cho et al. 2016). As shown in Figure 11a, strain CR29 exhibited the highest hydrophobicity at 25.88% ± 0.40%. The autoaggregation and coaggregation abilities of a strain are also crucial for its probiotic effects. Autoaggregation influences the adhesion of probiotics to intestinal epithelial cells, while coaggregation with pathogens can prevent these harmful bacteria from colonizing the gut (Gómez et al. 2017). Figure 11b presents the autoaggregation abilities of the nine LAB strains; the autoaggregation ability of CR29 was 16.72% ± 0.40%. The coaggregation abilities of the nine LAB strains with *Escherichia coli* and *Staphylococcus aureus* are shown in Figure 11c,d. JS75 and CR29 exhibited the highest coaggregation with *Escherichia coli* , with values of 22.20% ± 0.40% and 16.69% ± 0.50%, respectively. Similarly, JS75 and CR29 also showed the highest coaggregation abilities with *Staphylococcus aureus* , with coaggregation values of 17.99% ± 0.60% and 15.43% ± 0.30%, respectively.

In this study, CR29 demonstrated relatively high hydrophobicity, autoaggregation, and coaggregation abilities. Similarly, Ait Seddik et al. found that *Ligilactobacillus salivarius* p85lb1 and *Ligilactobacillus salivarius* p104lb1, among 67 LAB strains they isolated, exhibited similar properties (Ait Seddik et al. 2017). Kumar et al. also observed that *Ligilactobacillus salivarius* isolated from calf feces showed high autoaggregation and coaggregation abilities (Kumar et al. 2022), consistent with the results of this experiment.

#### 3.6.3 Survival Rate in a Simulated Gastrointestinal Environment

The survival rates of the nine LAB strains after treatment in a simulated artificial gastrointestinal environment are shown in Table 4. The initial viable cell counts ranged from 10.84 ± 0.05 to 12.92 ± 0.07 lg CFU/mL. CR29 demonstrated the highest overall tolerance. After gastric juice exposure, its cell count decreased minimally (from 12.92 ± 0.07 to 8.87 ± 0.09 lg CFU/mL), with a survival rate as high as 68.61% ± 0.40%, indicating significantly higher gastric juice tolerance compared to the other strains (*p* < 0.05). In contrast, CR39 was the least tolerant. After the simulated bile juice test, the survival of CR29 was 51.97% ± 0.60%. The intestinal juice had a smaller impact on the viable cell counts compared to gastric juice and bile, with an average reduction of about 1 log unit.

**TABLE 4:** Survival rate of strains in simulated gastrointestinal environment.

Strains	Initial (lg CFU/mL)	SGJ (lg CFU/mL)	Survival rate (%)	SBJ (lg CFU/mL)	Survival rate (%)	SIJ (lg CFU/mL)	Survival rate (%)
JS19	12.292 ± 0.034^b^	7.174 ± 0.042^d^	58.36 ± 0.002^cd^	5.040 ± 0.085^c^	51.79 ± 0.006^d^	4.999 ± 0.081^d^	40.67 ± 0.008^e^
JS21	12.765 ± 0.069^a^	7.568 ± 0.057^c^	59.29 ± 0.008^c^	5.960 ± 0.056^b^	46.69 ± 0.004^c^	5.806 ± 0.041^b^	45.49 ± 0.001^c^
JS24	11.901 ± 0.041^d^	6.772 ± 0.056^e^	56.90 ± 0.005^de^	6.007 ± 0.065^b^	50.47 ± 0.006^b^	5.604 ± 0.036^bc^	47.09 ± 0.004^bc^
CR29	12.922 ± 0.070^a^	8.866 ± 0.092^a^	68.61 ± 0.004^a^	6.716 ± 0.050^a^	51.97 ± 0.006^b^	6.184 ± 0.028^a^	47.86 ± 0.004^b^
CR39	11.012 ± 0.018^e^	6.061 ± 0.045^f^	55.04 ± 0.005^e^	4.594 ± 0.058^d^	41.72 ± 0.005^d^	3.990 ± 0.047^e^	36.23 ± 0.004^f^
JS42	10.844 ± 0.046^e^	7.092 ± 0.047^d^	65.40 ± 0.007^b^	6.037 ± 0.054^b^	55.67 ± 0.007^a^	5.706 ± 0.062^bc^	52.61 ± 0.004^a^
JS43	12.132 ± 0.049^bc^	8.183 ± 0.032^b^	67.46 ± 0.005^ab^	6.160 ± 0.031^b^	50.78 ± 0.005^b^	5.572 ± 0.057^c^	45.93 ± 0.004^c^
JS44	11.936 ± 0.053^cd^	7.212 ± 0.035^d^	60.42 ± 0.002^c^	6.025 ± 0.072^b^	50.47 ± 0.005^b^	5.083 ± 0.042^d^	42.58 ± 0.003^d^
JS75	12.195 ± 0.043^b^	8.053 ± 0.059^b^	66.03 ± 0.003^b^	6.091 ± 0.046^b^	49.95 ± 0.003^b^	4.975 ± 0.0318^d^	40.80 ± 0.004^de^

*Note:* Nine strains of lactic acid bacteria simulated gastrointestinal process, respectively (*n* = 3). Initially, nine strains of lactic acid bacteria before simulated gastrointestinal treatment. Different letters of the same column data indicated significant difference (*p* < 0.05), and the same letter label indicated that the difference was not significant (*p* > 0.05).

Abbreviations: SBJ, simulated bile juice; SGJ, simulated gastric juice; SIJ, simulated intestinal juice.

Resistance to the harsh conditions of the gastrointestinal tract is an important prerequisite for effective colonization of probiotics (Dicks and Botes 2010), and CR29 still has a relatively high survival rate after simulating the gastrointestinal tract, which is the same as the results of previous studies (Ait Seddik et al. 2017). *Ligilactobacillus salivarius* CPN60, isolated by Gupta et al. from calf feces, showed a high survival rate after passing through the gastrointestinal environment (Gupta et al. 2021), which was consistent with the results of this study. These results highlight CR29's potential as a probiotic candidate.

## 4 Conclusion

In this study, 16S rDNA high‐throughput sequencing was used to compare and analyze the microbiota between Jersey colostrum and mature milk. The results showed that the microbiota of mature milk had greater diversity, and there were significant differences in the distribution of core microbiota at the genus level. Additionally, nine strains of LAB with probiotic potential were isolated from Jersey milk. Experimental results indicated that *Ligilactobacillus salivarius* CR29 exhibited significant inhibitory effects against various pathogenic bacteria, was sensitive to multiple antibiotics, and had a high survival rate in low pH and high bile salt concentration environments, demonstrating excellent probiotic properties. This lays a foundation for further research on probiotics in Jersey milk.

## Author Contributions

**Qibin Wang:** designed and performed the experiments, drafted the manuscript. **Jianing Xu:** performed the experiment. **Lichun Shang:** provided the resources. **Qingshen Sun:** supervised the experiments, provided funding, reviewed the revised the final manuscript.

## Conflicts of Interest

The authors declare no conflicts of interest.

## Data Availability

All data are incorporated into the article and its online Supporting Information.
